# Surveillance and response for high-risk populations: what can malaria elimination programmes learn from the experience of HIV?

**DOI:** 10.1186/s12936-017-1679-1

**Published:** 2017-01-18

**Authors:** Jerry O. Jacobson, Carmen Cueto, Jennifer L. Smith, Jimee Hwang, Roly Gosling, Adam Bennett

**Affiliations:** 1 Malaria Elimination Initiative, Global Health Group, University of California, San Francisco, 550 16th Street, San Francisco, CA 94158 USA; 2 US President’s Malaria Initiative, Malaria Branch, Division of Parasitic Diseases and Malaria, US Centers for Disease Control and Prevention, 1600 Clifton Rd, Atlanta, GA 30333 USA

**Keywords:** Malaria, HIV, Surveillance, High risk populations

## Abstract

To eliminate malaria, malaria programmes need to develop new strategies for surveillance and response appropriate for the changing epidemiology that accompanies transmission decline, in which transmission is increasingly driven by population subgroups whose behaviours place them at increased exposure. Conventional tools of malaria surveillance and response are likely not sufficient in many elimination settings for accessing high-risk population subgroups, such as mobile and migrant populations (MMPs), given their greater likelihood of asymptomatic infections, illegal risk behaviours, limited access to public health facilities, and high mobility including extended periods travelling away from home. More adaptive, targeted strategies are needed to monitor transmission and intervention coverage effectively in these groups. Much can be learned from HIV programmes’ experience with “second generation surveillance”, including how to rapidly adapt surveillance and response strategies to changing transmission patterns, biological and behavioural surveys that utilize targeted sampling methods for specific behavioural subgroups, and methods for population size estimation. This paper reviews the strategies employed effectively for HIV programmes and offers considerations and recommendations for adapting them to the malaria elimination context.

## Background

As malaria programmes move from control to elimination, they need to reorient their surveillance and response systems to the changing epidemiology that accompanies transmission decline. A key challenge for reorienting surveillance and response systems is identifying and eliminating remaining reservoirs of infection that are increasingly geographically clustered [1, 2]. In addition to identifying geographic foci, many eliminating countries have identified distinct subpopulations at elevated risk of infection due to behaviours that increase their exposure to Anopheline mosquitoes [1–4]. These high-risk populations are thought to contribute disproportionately to sustaining transmission in low transmission areas and present challenges for reintroduction following elimination. They include groups that primarily acquire and transmit infection locally [5–7] as well as mobile and migrant populations (MMPs), which may import infections acquired elsewhere [1, 8–10].

Malaria control programmes typically rely on passive surveillance and national household surveys to monitor malaria burden and intervention coverage in the general population, with additional emphasis on young children and pregnant women due to their higher risk of severe disease. The establishment of a robust passive surveillance system remains fundamental for any malaria programme aiming for elimination, including a transition to case-based reporting and case investigation. However, as transmission becomes increasingly clustered in specific populations due to their behaviours, more targeted active surveillance and interventions are often required. These targeted surveillance strategies are needed in order to effectively identify specific high-risk behaviours and populations, determine their size, and track them over time with sufficient representativeness to accurately assess rates of infection, knowledge, and use of preventive measures, all of which are likely to evolve over time.

The passive surveillance systems that are currently the backbone of malaria surveillance globally are in many settings inadequate for the task of identifying and targeting high-risk populations for several reasons. First, individuals with the greatest exposure may be more likely to develop partial immunity resulting in asymptomatic or sub-clinical infection and, therefore, may be disproportionately less likely to present for care [11–15]. Second, many high-risk populations identified to date have limited access to public health facilities, which are central to passive surveillance [2, 10]. Third, even when high-risk individuals seek treatment, most passive surveillance systems do not gather the data that would be necessary to identify behavioural risk factors and effectively target behavioural risk groups. Fourth, in many contexts undocumented travel and illicit forest work are linked with increased risk; individuals may be hesitant to report these illicit activities without adequate confidentiality protections and appropriate questioning techniques. Finally, because MMPs and forest workers are frequently away from their households, active and reactive surveillance conducted through household visits may fail to capture them [10, 16].

While the context is distinct, HIV control programmes have faced similar challenges for some time. HIV transmission in most countries is focal or “concentrated” in high-risk populations, which face barriers to testing due to the illegality and stigma of the behaviours linked to increased risk and are not efficiently or effectively identified through household surveys [17–19]. Malaria can be characterized by acquired immunity or, like HIV, a long asymptomatic and infectious period, particularly in the case of *Plasmodium vivax* [20]. In HIV, these circumstances have led to distributions of reported cases that often do not reflect the relative contribution of different subpopulations to transmission or the current epidemiological situation [21].

This paper reviews strategies and best practices developed in the context of HIV for surveillance and response in high-risk populations and provides recommendations for adapting them, where appropriate, to malaria elimination settings.

### Search strategy and selection criteria

A literature review was conducted to identify evidence of high-risk populations in malaria by searching PubMed using the terms “malaria + elimination + high + risk + populations”. HIV literature was selected by reviewing the series of guidelines published by UNAIDS/WHO Working Group on Global HIV/AIDS and STI Surveillance [22] as well as studies illustrating approaches known to the authors.

### Populations at increased risk for malaria in elimination settings

In all settings, individual risk of malaria infection is determined by environmental factors that influence the density of competent anopheline mosquitos, location-specific vector behaviour, and human behavioural factors that increase an individual’s exposure to infectious bites. In many elimination settings, residual transmission exists due to anopheline species that exhibit outdoor feeding and resting behaviour (exophagy and exophily), which often coincides with outdoor human behaviours during biting hours [23, 24]. While long-lasting insecticide-treated nets (LLINs) and indoor residual spraying (IRS) are effective for more endophagic and endophilic anopheline species, where high coverage of LLINs and IRS has been achieved, programmes often observe behavioural shifts towards increased outdoor feeding and resting [25–27], as well as increased proportional abundance of more exophilic species such as *Anopheles arabiensis* [28]. In much of Asia, and southeast Asia in particular, malaria transmission persists due to forest-adapted vectors, such as *Anopheles dirus,* which exhibit outdoor biting behaviour, transmit *Plasmodium knowlesi* between macaque monkeys and humans, and are very difficult to control [29, 30].

Economic forces are a major factor responsible for human movement and bring people into closer contact with vectors through migration, forest work or specific livelihood activities. Table 1 summarizes some of the risk factors identified in countries moving from controlled low-endemic malaria to elimination (“malaria-eliminating” countries) [31] (Table 1). Several occupational groups have been identified as risk populations in different elimination settings, including laborers in fishing and agriculture, military, mining, construction, oil and gas, and general forest work. In southeast Asia, rubber plantation workers are a specific risk group predicted to increase in coming years, and are often highly mobile [32]; these groups and other migrant forest workers often have poor social integration due to language and cultural differences and occasionally, illegal activity [33]. Importation of cases by migrant labourers moving between high and low transmission settings, both within and between countries, can challenge elimination efforts. For example, seasonal farm work facilitates importation of cases between regions of Ethiopia [34]. Following elimination of local transmission in Sri Lanka, cases were imported by fishermen returning from Sierra Leone and military personnel returning from South Sudan and Haiti [8]. Cases have been imported to China by gold miners returning from Ghana [35]. Similarly, migrant laborers have frequently been found to be a key driver of incipient HIV epidemics [36].

**Table 1:** Population groups at elevated risk for malaria

Region/country	High-risk livelihood or occupational activities			Mobile populations	Demographic groups
	Forest	Agricultural	Mining/military/other		
South/East Asia					
Bhutan [3, 106]	Men collecting firewood	Men sleeping in the fields to protect crops		Businessmen travelling to India	
		Farmers			
Cambodia [5, 33, 107]	Male forest workers			Temporary migrants	Ethnic minority groups living on the forest fringe
					Jarai male youth
Indonesia [37]	Forest workers		Military personnel	Tourists	
			Miners	Migrants	
Malaysia [40, 108, 109]	Logging, fishing and other forest work	Plantation workers	Military personnel		Indigenous Orang Asli in the hinterland, particularly children
Sri Lanka [8, 110]	Adult men working on forest fringe		Adult male gem miners	Military personnel returning from South Sudan and Haiti	
				Fisherman returning from Sierra Leone	
				Laborers and migrants from Myanmar	
				Pakistani asylum seekers	
Thailand [46, 108, 109, 111–113]	Rubber tapers			Migrants from Myanmar, Cambodia and Laos	
				Internal migrants from rural areas to foothills or forests	
				Migrant workers	
				Mobile ethnic groups	
				Foreign travellers	
Vietnam [6, 114]	Forest-goers	Agricultural workers			Ra-glai population
	Male wood-cutters				Lower socioeconomic status
East Asia/Pacific					
China [115–118]				Laborers returning from Africa	
				Gold miners returning from Ghana	
				Laborers returning to Jiangsu from elsewhere in China	
Philippines [38, 39]	Forest clearing, logging, wood gathering, hunting		Military personnel		Indigenous populations, particularly in rural areas
			Charcoal workers		
			Construction workers		
North Korea [119]		Agricultural workers, ages 17–59 years	Industrial workers, ages 17–59 years		
Latin America					
Brazil [4, 120–123]			Adult male gold miners		
Suriname [124]				Illegal gold miners in remote areas	
Venezuela [125, 126]			Gold miners		
Other					
Saudi Arabia [127]				Adult males from North India	
				Migrants from southwest to east of country	
Swaziland [45]				Adult male migrant workers, primarily from Mozambique	
Turkmenistan [128]			Military personnel		Males in rural areas
			Oil and gas workers		
Ethiopia [34]				Young adult seasonal migrant farm workers in northwest Ethiopia	

Much remains unknown regarding how to best access the malaria risk populations identified to date for purposes of surveillance and response: the extent to which they may be captured by present surveillance and response activities; how to disseminate interventions with high coverage; and how to accurately track transmission and monitor intervention coverage. Some risk populations that have been more broadly defined—such as “tourists”, “foreign travellers” and “migrants” [37], “rural, indigenous populations” [1, 38, 39] and particular ethnic groups [6, 40]—require sharper definition to allow for meaningful targeting in specific elimination settings. Malaria programmes have begun to experiment with innovative strategies to access high-risk populations, often drawing on tools developed in the context of HIV; for example, screening at border crossings [41] and refugee camps [42], venue-based surveys [34], respondent-driven sampling (RDS) surveys [43, 44] and interviewing the social contacts of recent cases to identify behavioural risk factors [45]. Yet, such strategies have not yet solidified into routine practice. Improving surveillance and response for high-risk populations is a high priority for malaria eliminating countries, and is especially urgent in southeast Asia where MMPs are thought to influence the spread of artemisinin resistance [46].

While some similarities exist, HIV and malaria are characterized by distinct transmission mechanisms and risk factors that impact the effectiveness of potential surveillance and response approaches for high risk populations: malaria risk is determined by human-vector contact at specific times in geographic areas where specific vectors are endemic, while HIV is transmitted person-to-person primarily through sexual intercourse, drug injection, and blood transfusion. Nonetheless, both HIV programmes and malaria elimination programmes face the similar challenge of identifying and accessing relatively small high-risk populations that are key to continued transmission, which are at increased risk from specific behaviours or occupations, and yet which may be systematically missed by passive surveillance and household surveys. In the malaria context, these populations are often difficult to access due to cross-border mobility, high rates of asymptomatic infection, frequently being away from their home due to forest or other work, and poor social integration in the case of migrant workers. These factors additionally make it difficult to achieve high coverage of interventions and to accurately track trends in infection prevalence and prevention coverage.

### Learning from HIV “Second Generation Surveillance”

In HIV, guidelines for Second Generation Surveillance (SGS) released in 2000 were a response to these challenges [47, 48]. Data from “first-generation” HIV surveillance—case reporting and limited prevalence studies—were seen as inadequate to form a complete picture of transmission in the presence of hard-to-reach, high-risk populations.

**Fig. 1:**
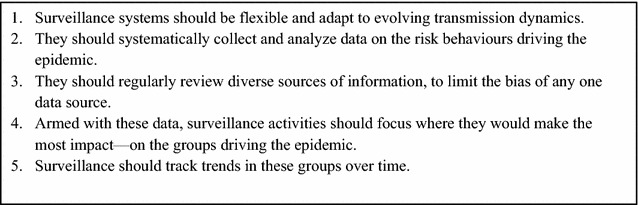
Principles of Second Generation Surveillance for HIV. Adapted from [48]

The SGS guidelines included a series of key principles to guide surveillance systems (Fig. 1). One change was expanding the scope of surveillance, in part by encouraging HIV programmes to examine a range of “markers” of potential risk, such as data on other sexually transmitted infections and tuberculosis. More relevant to malaria is the SGS principle that surveillance systems gather and analyze behavioural data. This is primarily done in two ways. First, passive surveillance expands to describe the distribution of risk behaviours among passively detected cases by recording whether they engage in known risk behaviours (e.g., sex work or, in malaria, forest work) on case report forms. Second, HIV programmes conduct behavioural surveys (these surveys are called "behavioural surveillance surveys" or BSS, because they are used for tracking trends over time) [49]. Behavioural surveys gather information on the target population’s knowledge and understanding of risk, the specific practices that lead to risk, the use of preventive measures, and treatment-seeking. Behavioural data thus allow for better understanding of how transmission is occurring, identify gaps in prevention coverage and treatment services, and provide early warning of upticks in transmission (if risk behaviours increase or prevention coverage declines). They can also help explain trends in case data and prevalence.

Many countries routinely conduct integrated biological and behavioural surveys (IBBS), which are similar to malaria indicator surveys (MIS), yet generally target a specific risk group, collect biological specimens to assess prevalence (and often incidence) and include a linked behavioural survey. SGS also includes studies to estimate the size of high-risk populations, often conducted together with BSS and IBBS to reduce costs [50]. BSS and IBBS utilize sampling methods appropriate to the target population, overcoming the chief limitation of passive and household surveillance to provide unbiased estimates among hard-to-reach, high-risk populations, for example, by sampling sex workers at sex work locations and through peer networks [51].

New tools have been introduced to assist countries synthesize data from these multiple sources in order to prioritize populations and locations and to define surveillance and intervention strategies; these include the pre-surveillance assessment (PSA) [52], public health triangulation [53], and integrated epidemiologic profile [54].

**Fig. 2:**
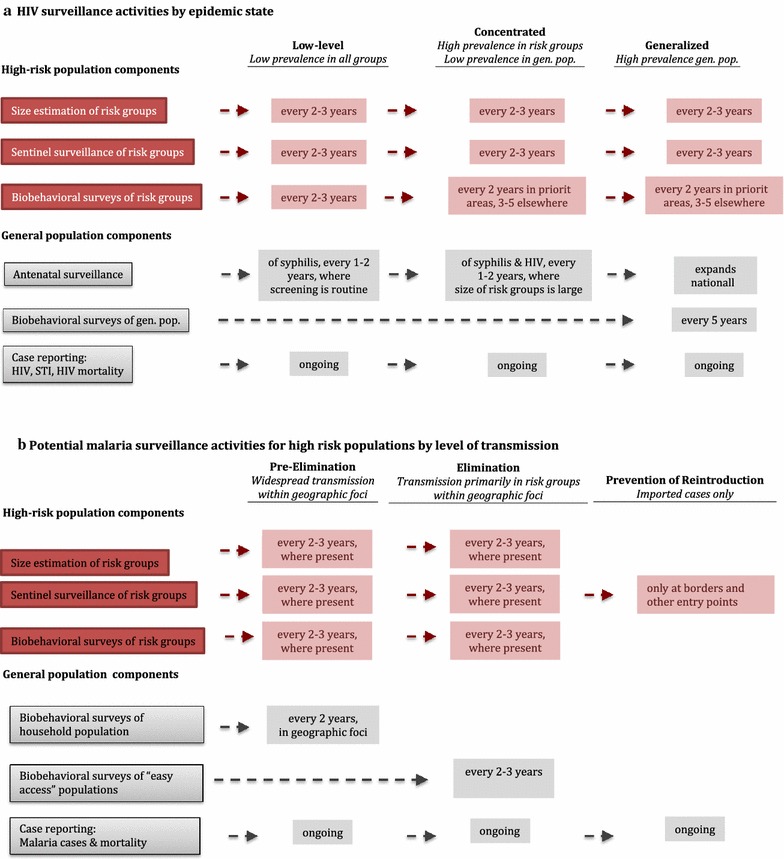
HIV and malaria surveillance activities for high-risk populations. Adapted from [49]; gen. pop., general population; STI, sexually transmitted infections

A key concept of SGS for HIV is that surveillance components should be adaptive to changing transmission patterns (Fig. 2a). For example, in countries where HIV transmission is primarily “concentrated” among high-risk populations, it is recommended that HIV programmes regularly conduct studies to estimate the size of these groups and IBBS in order to track prevalence of infection, intervention coverage, and to gather additional demographic and behavioural data to improve the targeting of interventions. In addition, sentinel surveillance is also recommended, typically at antenatal care (ANC) facilities, to provide early warning of expansion of transmission beyond high-risk groups. When HIV transmission has become widespread in the general population (“generalized” epidemics), it is recommended that countries expand ANC sentinel surveillance nationally and conduct IBBS in the general household population (i.e., by household surveys). Even in generalized epidemics, targeted surveillance of high-risk populations continues as targeted interventions can exert considerable impact [55, 56]. Regardless of the transmission pattern, countries should continually improve access to testing and passive surveillance.

### Tailoring malaria surveillance strategies for high-risk populations as transmission declines

Similar to HIV, at higher levels of transmission, malaria surveillance systems rely primarily on passive surveillance supplemented by large cross-sectional surveys such as an MIS. As transmission levels decline, the World Health Organization (WHO) recommends a shift in the scale of surveillance from reporting aggregate data on malaria morbidity and mortality at the health facility or district level to more rapid, finer scale case-based surveillance, which requires rapid reporting and investigation of individual cases and geographic foci. Detailed case and foci investigation is important for classifying cases as local or imported, determining the source of transmission, and planning responses. These changes are a necessary response to the changing epidemiology of malaria at low transmission, which is more likely to be imported, and occur more focally, seasonally and in older demographic groups. However, targeted surveillance of highly mobile high-risk populations continues to be limited within this framework. While reactive case detection (RCD) is frequently adopted to target active surveillance and response to areas with higher local transmission [1], other risk-based surveillance components to actively target high-risk populations are only rarely incorporated into malaria surveillance.

#### What lessons for tailoring malaria surveillance can be learned from SGS for HIV?

A potential adaption of HIV surveillance activities for malaria high risk populations as transmission declines is illustrated in Fig. 2b. In pre-elimination and elimination areas where some of the previously described malaria high-risk populations are present, targeted strategies are needed. Where such high-risk groups have been identified and are accessible, malaria programmes should consider conducting more targeted surveys in these groups to assess parasite prevalence and malaria risk behaviours, together with population size estimation. These surveys should be conducted in conjunction with entomological surveys to assess vector-specific risk behaviours. Additionally, targeted sentinel surveillance—i.e., data collection at existing services or programmes where a target population can be conveniently accessed—could be implemented at locations that serve or employ high-risk populations, such as clinics at military bases, logging and mining camps, or by screening returning foreign workers and migrant laborers at border checkpoints and agricultural and other worksites, as has been piloted in some settings [41, 57].

As countries move into the elimination phase, and transmission becomes increasingly clustered, it becomes less cost-effective to assess prevalence in the community using household-level surveys. Particularly where there are high rates of asymptomatic infection and/or barriers to testing, screening and surveys of “easy access” populations (i.e., at schools and ANC facilities) [58] may be considered as a potential general population comparison to high-risk groups, although potential biases should be evaluated. Targeted surveillance of high-risk populations should be intensified, prioritizing areas where they exist in large numbers such as worksites or known travel corridors. Following elimination, focus should shift to preventing reintroduction by maintaining a strong passive surveillance system, including vigilance and training of health workers to recognize symptoms of malaria, and surveillance of high-risk groups at borders and other entry points.

**Fig. 3:**
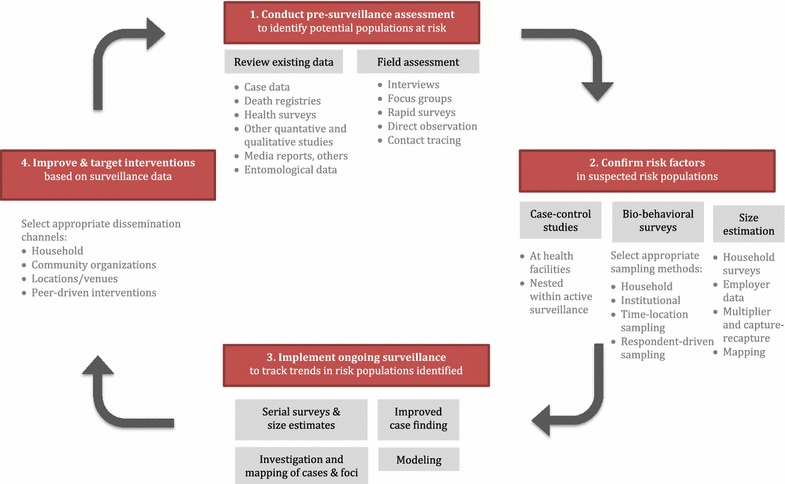
Surveillance cycle for targeting risk populations for malaria

To rapidly adapt as transmission patterns evolve, malaria programmes will need a mechanism to periodically reevaluate and reorient their high-risk population surveillance systems. In HIV SGS, this is accomplished through a four-step “surveillance cycle” [52]: (1) review available evidence to update understanding of transmission patterns and surveillance gaps; (2) confirm which populations are at risk through more rigorous studies; (3) adapt surveillance strategies as necessary; (4) refine interventions based on surveillance findings (Fig. 3).

### Establishing surveillance priorities through pre-surveillance assessment

The first step of the surveillance cycle is to assess the overall surveillance system’s strengths and weaknesses, based on current understanding of how and where transmission is occurring, and reorient surveillance strategies as needed. Key questions include: Which population subgroups, in which areas, are most affected?; Which are key drivers of transmission?; Are these subgroups adequately captured and characterized by existing surveillance activities (e.g., passive surveillance, prevalence and behavioural surveys, sentinel surveillance)?; If not, what new data and strategies are needed? In HIV this assessment is called “pre-surveillance assessment” (PSA) because it is generally undertaken prior to planning a new round of targeted surveillance studies nationally [52]. PSA could be a valuable tool for malaria elimination programmes as they endeavor to identify and address high-risk populations. In malaria contexts, PSA could result in decisions regarding how to strengthen passive surveillance (e.g., adding behaviourial data items to case reporting forms, engaging more private sector providers in reporting), identifying new high-risk populations where targeted studies and/or size estimates are needed, and, overall, determining which populations and areas should be prioritized by surveillance to achieve greatest impact.

A coordinating body (often organized as a working or advisory group) is often established to guide the PSA process due to the many actors typically involved in supporting prevention and surveillance activities in HIV high-risk populations. Although surveillance decisions are ultimately made by a unit in the health ministry, the coordinating body helps to ensure the data collected by the surveillance system meet the country’s needs, ensures widespread use of data, and makes certain the system is adequately funded. Malaria programmes may find such a structure helpful as they work increasingly with MMPs and occupational groups at elevated risk. During PSA, the coordinating body can help ensure the scope of the assessment is sufficiently wide-reaching, improve access to key informants and data sources, and help interpret the data.

**Fig. 4:**
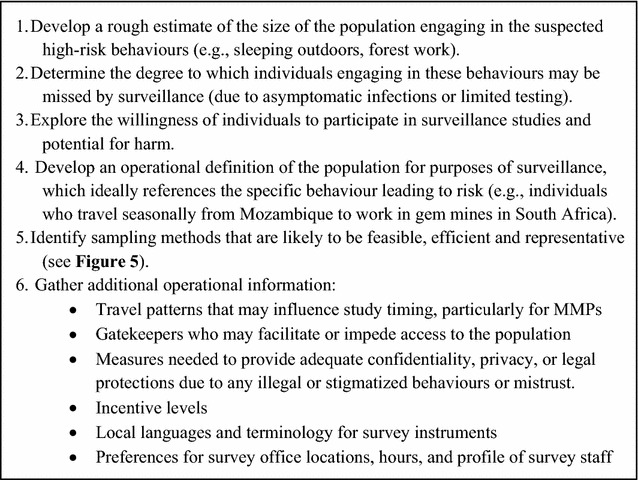
Aims for field assessment for planning targeted studies

Formative assessment—a narrower process to gather information needed to plan one particular study [59]—is typically conducted as part of the PSA as it becomes clear that targeted studies may be needed. PSA’s more high-level questions about prioritizing populations and determining surveillance strategies, as well as the narrower operational questions for study planning, are answered in part by a rapid “on-the-ground” field assessment, which includes key informant interviews, focus groups, mapping of risk contexts and other qualitative data collection methods. Key questions for field assessment include: Is the high-risk population large enough to warrant a targeted study? How should the population be defined for surveillance purposes? What sampling method is most appropriate? Other themes appear in Fig. 4.

As in HIV, malaria programmes can benefit from a wide-reaching PSA process. The data review should draw on all sources of information that could provide insight on populations potentially at elevated malaria risk, beginning from passive surveillance, but also considering health facility records, death registries, demographic health surveys, entomological surveillance data, vector control and other programme data from governmental and non-governmental organizations (NGOs) (e.g., military, agricultural, mining, logging) that work with the potential high-risk groups, and available qualitative studies and media reports to build a more complete picture of transmission and risk. A recent effort to characterize risk among MMPs in Cambodia provides a good example of data synthesis by drawing on diverse data sources [33, 60]. A recent WHO manual describes data sources and data collection methods that may be relevant to MMPs in the Greater Mekong Subregion [61]. Similarly, field assessment should query a broad spectrum of actors, such as community leaders, organizations with knowledge of the populations of interest, health workers, and members of the suspected risk populations, thus providing an opportunity to engage with affected communities. Contact tracing of malaria cases who report recent local travel or forest work can also help to develop hypotheses regarding behavioural risk factors and identify other individuals with whom they travelled or worked [45].

### Confirming risk factors and characterizing high-risk groups

Following PSA, more rigorous studies to estimate the prevalence of malaria parasite infection in suspected risk populations will likely be needed because evidence from PSA tends to be based on secondary analysis and qualitative methods. Case–control studies can effectively identify risk factors and are recommended by the WHO for malaria elimination settings with low case numbers [16, 62–65]. However, because case–control studies typically include individuals diagnosed at health facilities or sentinel sites, inference may be limited to symptomatic malaria. Patterns in service utilization may also introduce bias (e.g., if MMPs have limited access to public facilities). To overcome these limitations, case–control studies could be nested within active surveillance, such as RCD or in the context of a targeted parasite survey.

In HIV, IBBS are the standard tool to assess infection prevalence and behaviours in high-risk populations and to track these over time given high rates of asymptomatic infection barriers to testing [22]. Estimates of infection prevalence from IBBS are compared to a general population estimate to establish evidence of increased risk. For malaria, this could be done by comparison to targeted household surveys in potentially high-risk areas, potentially reducing costs by using lot quality assurance sampling, or by comparison to easy access groups [66].

#### Sampling methods for high-risk population surveys

**Fig. 5:**
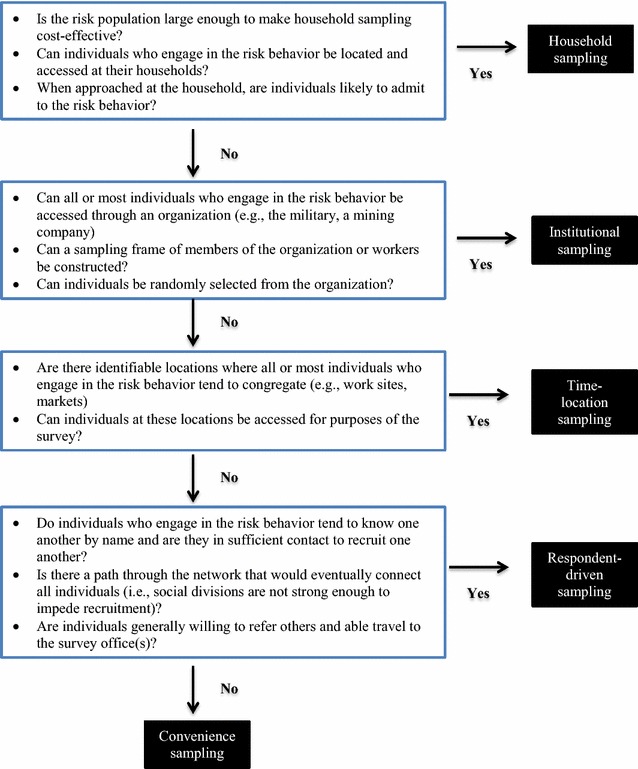
Flow diagram for selecting sampling methods for surveys in high-risk populations

Malaria programmes need to determine the most appropriate sampling method for each high-risk population identified. Figure 5 presents a flow diagram for selecting among sampling methods. Methods should be selected to achieve high representativeness, be acceptable to the affected population and feasible within a relatively short time frame (e.g., in HIV 3–4 months) [22]. The limitation of convenience sampling is that it produces findings that do not reflect the larger population and limits the ability to assess time trends.

Household-based sampling may be appropriate if individuals engaged in risk behaviours comprise a large fraction of the population and can be accessed at their households, such as in villages where forest work is common and individuals engaging in forest work are home at predictable days and times. Institutional sampling may prove more efficient when risk derives from the activities of a large organization, such as the military or a company.

When the risk population is “hidden” or “hard-to-reach”, that is, where conventional sampling fails because the population is relatively small, has privacy concerns, or a sampling frame of individuals cannot be constructed, then time-location sampling (TLS) or RDS may be appropriate [18, 22]. TLS begins by constructing a sampling frame of locations (“venues”) and times when the population at risk tends to congregate. Such locations in malaria might include work sites, mining and forest work camps, military bases, markets, bars, truck stops, and border crossings. To improve representativeness, TLS individuals meeting eligibility criteria are selected at random from randomly selected venues at randomly selected time intervals. Important considerations for TLS include whether a reasonably complete (and unbiased) sampling frame can be constructed, whether venues pose a safety risk to survey staff, and the extent to which venue-goers are representative of the larger risk population. In HIV, an example of bias is that TLS under-represents men who have sex with men (MSM) who tend not to frequent gay-identified locations, who often differ in their risk profile [67–69]. In malaria, TLS has been used to identify greater incidence of fever and reduced treatment-seeking among individuals who frequent bars and evening church services in Namibia [70], and higher parasite prevalence among seasonal migrant farm workers in Ethiopia recruited from farms, roads and town locations where migrant workers gather [34].

**Fig. 6:**
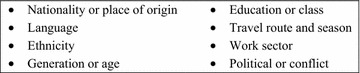
Potential sources of social fragmentation among MMPs. Adapted from [77]

RDS samples individuals by chain referral and is most effective when the individuals who engage in risk behaviours tend to know one another and are willing and able to travel to one or more designated survey offices [59, 71–73]. Representativeness of estimates produced by RDS can be compromised when the risk population does not comprise one well-connected network but instead is divided along characteristics that may be linked to risk; for example, HIV surveys of MSM often end up recruiting either high socioeconomic (SES) or low SES MSM, but not both [74]. With regard to MMPs, some HIV surveys using RDS have performed poorly due to social divisions owing to language, ethnicity, generational gaps, and other factors (Fig. 6) [75]. PSA or formative research should aim to identify such divisions. Selecting diverse “seeds” (first participants) can help to bridge the gaps when ties among subgroups are weak. When fragmentation is severe, RDS surveys should be limited to the most relevant well-connected subgroups [76]. In Thailand, two separate RDS surveys were undertaken to characterize migrant workers from Cambodia and Myanmar, respectively [43, 44].

RDS and TLS surveys require larger sample size than household surveys due to larger design effects [77, 78]. Analysis of RDS data requires specialized software [79] and diagnostic tests to assess whether theoretical assumptions were met during data collection [76].

#### Population size estimation

Estimates of the number of individuals with risk behaviours of interest are essential to advocate for resources, prioritize among groups, predict the impact of transmission via models, plan interventions, and assess programme coverage [50]. Population size estimation (PSE) is, therefore, integral to both surveillance and monitoring and evaluation.

Size estimates derived from household surveys are often viewed as under-estimates in HIV because stigmatized risk behaviours may be under-reported due to being interviewed at the household, often together with other householders. In malaria, household surveys can be used to gauge the size of the population frequently outside during mosquito biting hours and of subgroups engaged in different risk activities. To reduce bias due to high-risk household members who tend to be absent from the household, household surveys should query respondents about all household members.

When behaviours of interest are illegal or stigmatized, PSE methods appropriate for hidden populations are necessary. The most common are mapping, capture-recapture and multiplier methods [50, 80–83]. Mapping estimates are derived by enumerating locations where the population congregates and recording the number of individuals present at a census or sample of these locations; they are often conducted in conjunction with TLS surveys. Mapping requires adjustments to account for individuals absent during observation, attendance patterns, and double-counting.

Capture-recapture and multiplier size estimates involve comparison of multiple samples of the target population, one of which must be random. Samples commonly derive from IBBS surveys (as the random sample), records of services or community groups (for “service multipliers”), and distributing objects to accessible population members (for “unique object multipliers”) [81, 82]. Samples must be independent and the population must be stable between captures, potentially limiting the utility of these methods for mobile populations. Programmes should aim to conduct and triangulate multiple size estimates due to the potential for bias and low precision of available methods [83].

### Ongoing surveillance of high-risk populations

#### Serial surveys and size estimates

Once high-risk populations have been identified, HIV programmes generally conduct targeted surveys and size estimates every 2–5 years, depending on context [22, 49, 50], providing the strategic information necessary to adapt interventions as conditions evolve. These activities should be repeated often enough to detect changes relevant to the design of interventions in each high-risk population. To enable assessment of trends, malaria programmes should select methods that can be reliably repeated over time. Once elimination is achieved, surveys can be replaced as discussed earlier, by increased vigilance and screening at borders and entry points.

#### Improving case finding

Case finding can also be improved to be made more sensitive to high-risk populations. For example, case investigation forms should be updated to track any known high-risk behaviours. Geographic mapping of cases, high-risk locations, and remote transmission foci, if feasible, can help improve targeting of interventions [84].

As part of routine surveillance, many malaria elimination programmes conduct household-based RCD, which involves testing and treating individuals in close geographic proximity to an index case. However, there is limited evidence to guide how household-based RCD is conducted and whether it has any impact on transmission [85–87]. Where risk is driven more by behaviours than by household location, malaria programmes could adapt RCD to instead screen and treat social contacts of the index case who engage in known risk behaviours, such as forest work colleagues, as a form of “socio-behavioural RCD”. For example, HIV programmes have improved case detection by enlisting cases to refer potentially high-risk peers to testing services [88, 89].

#### Transmission models

In HIV, standardized transmission models developed by UNAIDS and WHO are applied biennially to update national and global estimates and projections, drawing on countries’ most recent surveys, size estimates and other data [90–92]. These models are a central feature of HIV surveillance systems that allow countries to estimate the relative contribution of high-risk populations to new infections, predict transmission patterns over time and assess the potential impact of new interventions. In settings where high-risk populations may contribute disproportionately to transmission, adapting malaria transmission models to account for them could provide valuable insight on the potential impact of control strategies that target individuals at greatest risk [93].

### Targeting interventions

Achieving appropriate targeting and high levels of coverage are often the key challenges of interventions in high-risk populations [94]. To improve trust, acceptance and effectiveness, engaging with communities is critical to HIV interventions. Planning should include identifying community organizations and leaders, which often already have the know-how to access risk populations, and may hold influence on the population’s receptiveness to new initiatives. Including members of the target population as project coordinators, interviewers, trainers, health or peer educators is a best practice in HIV. At the same time, those planning interventions should be aware of divisions when affiliating with any particular group or individual.

**Fig. 7:**
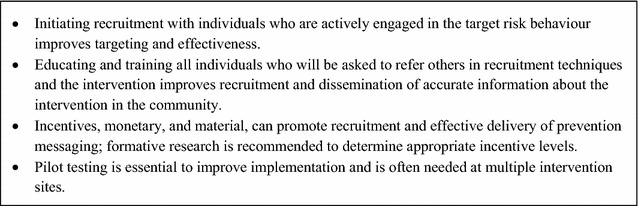
Lessons learned for peer-driven interventions

Malaria programmes’ conventional approaches to disseminating interventions—through household visits, health facilities, and community health workers—may be effective when risk and/or risk behaviours are widespread, and high-risk individuals tend to develop symptomatic infections, are accessible at their households and have good access to health care. If instead risk clusters within workers of an organization, then offering workers regular access to prevention, screening, and treatment may be more efficient. To extend reach beyond the often small subset of individuals linked with community organizations, HIV interventions have adopted approaches that parallel sampling strategies. For example, venue-based outreach often begins by mapping of risk locations [95, 96]. As in RDS, peer-driven interventions (PDIs) enlist high-risk individuals to provide prevention information to their social contacts [97], thus harnessing trust and peer pressure to expand reach, and often employing incentives to improve participation and referral [98, 99]. Social network strategies have proven successful outside of HIV; for example, exposing nominated friends of random villagers to a nutritional programme increased communities’ overall uptake compared to targeting random villagers [100]. Venue-based and PDI approaches have also been combined effectively, by initiating peer-referral with individuals encountered at high-risk venues [101]. In malaria, peer educators could be enlisted to disseminate information and/or preventive items and refer peers to testing and prevention programmes; dissemination of larger commodities, such as LLINs, poses logistical challenges but could be explored. Lessons learned regarding PDIs in the context of HIV [97–99, 102–105] are shown in Fig. 7.

## Conclusion

Malaria programmes embarking upon elimination must first draw on diverse sources of data to clearly define the subgroups most likely to be at elevated risk through pre-surveillance assessment, conduct serial targeted surveys, case–control studies, and/or sentinel surveillance to confirm risk, determine the approximate size of risk groups to guide prioritization and planning of interventions, and adopt a surveillance cycle to ensure that surveillance and response activities adapt as transmission patterns evolve.

There may be fewer challenges to conducting surveillance of high-risk populations in the malaria context relative to HIV. First, malaria and malaria risk groups, perhaps with the exception of some MMPs, are generally not stigmatized. Second, in general, malaria infections are shorter in duration, so that behavioural data will be more closely linked to infection risk in time, which should facilitate the identification of risk factors. Third, malaria is curable and treatment is both more brief and less expensive than in HIV, providing greater motivation to participate in surveys that provide free testing. Where HIV and malaria populations coincide (e.g., some mobile populations may be at risk for both), harmonizing surveillance and response activities may provide a way to reduce costs.

## Abbreviations

ANC: antenatal care
BSS: behavioural surveillance survey
HIV: human immunodeficiency syndrome
IBBS: integrated biological and behavioural survey
IRS: indoor residual spraying
LLIN: long-lasting insecticide-treated net
MIS: malaria indicator survey
MMP: mobile and migrant population
MSM: men who have sex with men
NGO: non-governmental organization
PDI: peer-driven intervention
PSA: pre-surveillance assessment
PSE: population size estimate or estimation
RCD: reactive case detection
RDS: respondent-driven sampling
SGS: second generation surveillance
TLS: time-location sampling
WHO: World Health Organization
UNAIDS: United Nations Programme on HIV/AIDS

## References

[1] Cotter C, Sturrock HJW, Hsiang MS, Liu J, Phillips AA, Hwang J (2013). The changing epidemiology of malaria elimination: new strategies for new challenges. Lancet.

[2] Sturrock HJ, Hsiang MS, Cohen JM, Smith DL, Greenhouse B, Bousema T (2013). Targeting asymptomatic malaria infections: active surveillance in control and elimination. PLoS Med..

[3] Yangzom T, Gueye CS, Namgay R, Galappaththy GN, Thimasarn K, Gosling R (2012). Malaria control in Bhutan: case study of a country embarking on elimination. Malar J.

[4] Barbieri AF, Sawyer DO (2007). Heterogeneity of malaria prevalence in alluvial gold mining areas in Northern Mato Grosso State, Brazil. Cad Saude Publica.

[5] Dysoley L, Kaneko A, Eto H, Mita T, Socheat D, Börkman A (2008). Changing patterns of forest malaria among the mobile adult male population in Chumkiri District, Cambodia. Acta Trop.

[6] Grietens KP, Xuan XN, Van Bortel W, Duc TN, Ribera JM, Nhat TB (2010). Low perception of malaria risk among the Ra-glai ethnic minority in south-central Vietnam: implications for forest malaria control. Malar J.

[7] Moreno J, Rubio-Palis Y, Páez E, Pérez E, Sánchez V (2007). Abundance, biting behaviour and parous rate of anopheline mosquito species in relation to malaria incidence in gold-mining areas of southern Venezuela. Med Vet Entomol.

[8] Dharmawardena P, Premaratne RG, de AW Gunasekera WKT, Hewawitarane M, Mendis K, Fernando D (2015). Characterization of imported malaria, the largest threat to sustained malaria elimination from Sri Lanka. Malar J.

[9] Pindolia DK, Garcia AJ, Wesolowski A, Smith DL, Buckee CO, Noor AM (2012). Human movement data for malaria control and elimination strategic planning. Malar J.

[10] Wangdi K, Gatton ML, Kelly GC, Clements AC (2015). Cross-border malaria: a major obstacle for malaria elimination. Adv Parasitol.

[11] Macauley C (2005). Aggressive active case detection: a malaria control strategy based on the Brazilian model. Soc Sci Med.

[12] Alves FP, Durlacher RR, Menezes MJ, Krieger H, Silva LHP, Camargo EP (2002). High prevalence of asymptomatic *Plasmodium vivax* and *Plasmodium falciparum* infections in native Amazonian populations. Am J Trop Med Hyg.

[13] Pinto J, Sousa CA, Gil V, Ferreira C, Gonçalves L, Lopes D (2000). Malaria in Sao Tomé and Prıncipe: parasite prevalences and vector densities. Acta Trop.

[14] Park CG, Chwae Y-J, Kim J-I, Lee J-H, Hur GM, Jeon BH (2000). Serologic responses of Korean soldiers serving in malaria-endemic areas during a recent outbreak of *Plasmodium vivax*. Am J Trop Med Hyg.

[15] Laishram DD, Sutton PL, Nanda N, Sharma VL, Sobti RC, Carlton JM (2012). The complexities of malaria disease manifestations with a focus on asymptomatic malaria. Malar J.

[16] WHO (2012). Disease surveillance for malaria elimination: an operational manual.

[17] Joint United Nations Program on HIV/AIDS. Global report: UNAIDS report on the global AIDS epidemic 2013; 2013.

[18] Magnani R, Sabin K, Saidel T, Heckathorn D (2005). Review of sampling hard-to-reach and hidden populations for HIV surveillance. AIDS.

[19] Heckathorn D (1997). Respondent-driven sampling: a new approach to the study of hidden populations. Soc Probl.

[20] Kevin Baird J (2013). Malaria caused by *Plasmodium vivax*: recurrent, difficult to treat, disabling, and threatening to life—averting the infectious bite preempts these hazards. Pathog Glob Health.

[21] Mishra S, Pickles M, Blanchard JF, Moses S, Boily M-C (2014). Distinguishing sources of HIV transmission from the distribution of newly acquired HIV infections: why is it important for HIV prevention planning?. Sex Transm Infect.

[22] WHO, Joint United Nations Programme on HIV/AIDS (2011). Guidelines on surveillance among populations most at risk for HIV.

[23] Killeen GF (2014). Characterizing, controlling and eliminating residual malaria transmission. Malar J.

[24] Monroe A, Asamoah O, Lam Y, Koenker H, Psychas P, Lynch M (2015). Outdoor-sleeping and other night-time activities in northern Ghana: implications for residual transmission and malaria prevention. Malar J.

[25] Moiroux N, Gomez MB, Pennetier C, Elanga E, Djenontin A, Chandre F (2012). Changes in *Anopheles funestus* biting behavior following universal coverage of long-lasting insecticidal nets in Benin. J Infect Dis.

[26] Padonou GG, Gbedjissi G, Yadouleton A, Azondekon R, Razack O, Oussou O (2012). Decreased proportions of indoor feeding and endophily in *Anopheles gambiae* s.l. populations following the indoor residual spraying and insecticide-treated net interventions in Benin (West Africa). Parasit Vectors.

[27] Meyers JI, Pathikonda S, Popkin-Hall ZR, Medeiros MC, Fuseini G, Matias A (2016). Increasing outdoor host-seeking in *Anopheles gambiae* over 6 years of vector control on Bioko Island. Malar J.

[28] Sinka ME, Golding N, Massey NC, Wiebe A, Huang Z, Hay SI (2016). Modelling the relative abundance of the primary African vectors of malaria before and after the implementation of indoor, insecticide-based vector control. Malar J.

[29] Hii J, Rueda LM (2013). Malaria vectors in the Greater Mekong Subregion: overview of malaria vectors and remaining challenges. Southeast Asian J Trop Med Public Health.

[30] Van Bortel W, Trung HD, le Hoi X, Van Ham N, Van Chut N, Luu ND (2010). Malaria transmission and vector behaviour in a forested malaria focus in central Vietnam and the implications for vector control. Malar J.

[31] Newby G, Bennett A, Larson E, Cotter C, Shretta R, Phillips A (2016). The path to eradication: a progress report on the malaria-eliminating countries. Lancet.

[32] Tangena JA, Thammavong P, Wilson AL, Brey PT, Lindsay SW (2016). Risk and control of mosquito-borne diseases in Southeast Asian rubber plantations. Trends Parasitol.

[33] Grietens KP, Gryseels C, Dierickx S, Bannister-Tyrrell M, Trienekens S, Uk S (2015). Characterizing types of human mobility to inform differential and targeted malaria elimination strategies in Northeast Cambodia. Sci Rep.

[34] Schicker RS, Hiruy N, Melak B, Gelaye W, Bezabih B, Stephenson R (2015). A venue-based survey of malaria, anemia and mobility patterns among migrant farm workers in Amhara Region, Ethiopia. PLoS ONE.

[35] Sturrock HJ, Roberts KW, Wegbreit J, Ohrt C, Gosling RD (2015). Tackling imported malaria: an elimination endgame. Am J Trop Med Hyg.

[36] Deane KD, Parkhurst JO, Johnston D (2010). Linking migration, mobility and HIV. Trop Med Int Health.

[37] Lederman ER, Sutanto I, Wibudi A, Ratulangie L, Rudiansyah I, Fatmi A (2006). Imported malaria in Jakarta, Indonesia: passive surveillance of returned travelers and military members postdeployment. J Travel Med.

[38] Lansang MAD, Belizario V, Bustos M, Saul A, Aguirre A (1997). Risk factors for infection with malaria in a low endemic community in Bataan, the Philippines. Acta Trop.

[39] Ministry of Health Philippines, WHO, University of California (2014). Eliminating malaria: case-study 6—progress towards subnational elimination in the Philippines.

[40] Kaur G (2009). Malaria endemicity in an Orang Asli community in Pahang, Malaysia. Trop Biomed..

[41] Edwards HM, Canavati SE, Rang C, Ly P, Sovannaroth S, Canier L (2015). Novel cross-border approaches to optimise identification of asymptomatic and artemisinin-resistant Plasmodium infection in mobile populations crossing Cambodian Borders. PLoS ONE.

[42] Carrara VI, Sirilak S, Thonglairuam J, Rojanawatsirivet C, Proux S, Gilbos V (2006). Deployment of early diagnosis and mefloquine–artesunate treatment of falciparum malaria in Thailand: the Tak Malaria Initiative. PLoS Med.

[43] Khamsiriwatchara A, Wangroongsarb P, Thwing J, Eliades J, Satimai W, Delacollette C (2011). Respondent-driven sampling on the Thailand–Cambodia border. I. Can malaria cases be contained in mobile migrant workers. Malar J.

[44] Wangroongsarb P, Satimai W, Khamsiriwatchara A, Thwing J, Eliades JM, Kaewkungwal J (2011). Respondent-driven sampling on the Thailand–Cambodia border. II. Knowledge, perception, practice and treatment-seeking behaviour of migrants in malaria endemic zones. Malar J.

[45] Koita K, Novotny J, Kunene S, Zulu Z, Ntshalintshali N, Gandhi M (2013). Targeting imported malaria through social networks: a potential strategy for malaria elimination in Swaziland. Malar J.

[46] Carrara VI, Lwin KM, Phyo AP, Ashley E, Wiladphaingern J, Sriprawat K (2013). Malaria burden and artemisinin resistance in the mobile and migrant population on the Thai–Myanmar border, 1999–2011: an observational study. PLoS Med.

[47] Rehle T, Lazzari S, Dallabetta G, Asamoah-Odei E (2004). Second-generation HIV surveillance: better data for decision-making. Bull World Health Organ.

[48] WHO, Joint United Nations Programme on HIV/AIDS (2000). Guidelines for Second Generation HIV Surveillance.

[49] WHO, Joint United Nations Programme on HIV/AIDS (2013). Guidelines for second generation HIV surveillance: an update: know your epidemic.

[50] WHO, Joint United Nations Programme on HIV/AIDS (2010). Guidelines on estimating the size of populations most at risk to HIV.

[51] Ghys PD, Jenkins C, Pisani E (2001). HIV surveillance among female sex workers. AIDS.

[52] WHO, Joint United Nations Programme on HIV/AIDS (2005). The pre-surveillance assessment: guidelines for planning serosurveillance of HIV, prevalence of sexually transmitted infections and the behavioural components of second generation surveillance of HIV.

[53] Rutherford GW, McFarland W, Spindler H, White K, Patel SV, Aberle-Grasse J (2010). Public health triangulation: approach and application to synthesizing data to understand national and local HIV epidemics. BMC Public Health.

[54] Whitmore SK, Zaidi IF, Dean HD (2005). The integrated epidemiologic profile: using multiple data sources in developing profiles to inform HIV prevention and care planning. AIDS Educ Prev.

[55] Steen R, Hontelez JA, Veraart A, White RG, de Vlas SJ (2014). Looking upstream to prevent HIV transmission: can interventions with sex workers alter the course of HIV epidemics in Africa as they did in Asia?. AIDS.

[56] Boily M-C, Pickles M, Alary M, Baral S, Blanchard J, Moses S (2015). What really is a concentrated HIV epidemic and what does it mean for West and Central Africa? Insights from mathematical modeling. J Acquir Immune Defic Syndr.

[57] Fernando SD, Dharmawardana P, Semege S, Epasinghe G, Senanayake N, Rodrigo C (2016). The risk of imported malaria in security forces personnel returning from overseas missions in the context of prevention of re-introduction of malaria to Sri Lanka. Malar J.

[58] van Eijk AM, Hill J, Noor AM, Snow RW, ter Kuile FO (2015). Prevalence of malaria infection in pregnant women compared with children for tracking malaria transmission in sub-Saharan Africa: a systematic review and meta-analysis. Lancet Global Health.

[59] Johnston LG, Whitehead S, Simic-Lawson M, Kendall C (2010). Formative research to optimize respondent-driven sampling surveys among hard-to-reach populations in HIV behavioral and biological surveillance: lessons learned from four case studies. AIDS Care.

[60] Guyant P, Canavati SE, Chea N, Ly P, Whittaker MA, Roca-Feltrer A (2015). Malaria and the mobile and migrant population in Cambodia: a population movement framework to inform strategies for malaria control and elimination. Malar J.

[61] WHO (2015). Decision-tree framework for selecting study methods for malaria interventions in mobile and migrant populations.

[62] Yukich JO, Taylor C, Eisele TP, Reithinger R, Nauhassenay H, Berhane Y (2013). Travel history and malaria infection risk in a low-transmission setting in Ethiopia: a case control study. Malar J.

[63] Alexander N, Rodríguez M, Pérez L, Caicedo JC, Cruz J, Prieto G (2005). Case-control study of mosquito nets against malaria in the Amazon region of Colombia. Am J Trop Med Hyg.

[64] Osorio L, Todd J, Bradley DJ (2004). Travel histories as risk factors in the analysis of urban malaria in Colombia. Am J Trop Med Hyg.

[65] Lynch CA, Bruce J, Bhasin A, Roper C, Cox J, Abeku TA (2015). Association between recent internal travel and malaria in Ugandan highland and highland fringe areas. Trop Med Int Health.

[66] Robertson SE, Valadez JJ (2006). Global review of health care surveys using lot quality assurance sampling (LQAS), 1984–2004. Soc Sci Med.

[67] Paz-Bailey G, Miller W, Shiraishi RW, Jacobson JO, Abimbola TO, Chen SY (2013). Reaching men who have sex with men: a comparison of respondent-driven sampling and time-location sampling in Guatemala City. AIDS Behav.

[68] Kendall C, Kerr LR, Gondim RC, Werneck GL, Macena RHM, Pontes MK (2008). An empirical comparison of respondent-driven sampling, time location sampling, and snowball sampling for behavioral surveillance in men who have sex with men, Fortaleza, Brazil. AIDS Behav.

[69] Raymond HF, Rebchook G, Curotto A, Vaudrey J, Amsden M, Levine D (2010). Comparing internet-based and venue-based methods to sample MSM in the San Francisco Bay Area. AIDS Behav.

[70] Jacobson JO, Cueto C, Smith J, Mumbengegwi D, Roberts K, Sturrock H, et al. Evaluating high-risk venue-based malaria surveillance using time-location sampling in Namibia. In: American Society for Tropical Medicine & Hygiene; Philadelphia. Abstract; 2015.

[71] Toledo L, Codeco CT, Bertoni N, Albuquerque E, Malta M, Bastos FI (2011). Putting respondent-driven sampling on the map: insights from Rio de Janeiro, Brazil. J Acquir Immune Defic Syndr.

[72] McCreesh N, Johnston LG, Copas A, Sonnenberg P, Seeley J, Hayes RJ (2011). Evaluation of the role of location and distance in recruitment in respondent-driven sampling. Int J Health Geogr.

[73] Silva-Santisteban A, Raymond HF, Salazar X, Villayzan J, Leon S, McFarland W (2012). Understanding the HIV/AIDS epidemic in transgender women of Lima, Peru: results from a sero-epidemiologic study using respondent driven sampling. AIDS Behav.

[74] Kerr LR, Mota RS, Kendall C, Pinho AdA, Mello MB, Guimaraes MD (2013). HIV among MSM in a large middle-income country. AIDS..

[75] Tyldum G, Johnston L (2014). Applying respondent driven sampling to migrant populations: lessons from the field.

[76] Gile KJ, Johnston LG, Salganik MJ (2015). Diagnostics for respondent-driven sampling. J R Stat Soc Ser A Stat Soc.

[77] Johnston LG, Chen Y-H, Silva-Santisteban A, Raymond HF (2013). An empirical examination of respondent driven sampling design effects among HIV risk groups from studies conducted around the world. AIDS Behav.

[78] Gile KJ, Handcock MS (2010). Respondent-driven sampling: an assessment of current methodology. Sociol Methodol.

[79] Handcock MS, Fellows IE, Gile KJ. RDS analyst: software for the analysis of respondent-driven sampling data, version 0.42. 2014. http://hpmrg.org.

[80] Zhang D, Lv F, Wang L, Sun L, Zhou J, Su W (2007). Estimating the population of female sex workers in two Chinese cities on the basis of the HIV/AIDS behavioural surveillance approach combined with a multiplier method. Sex Transm Infect.

[81] Johnston LG, Prybylski D, Raymond HF, Mirzazadeh A, Manopaiboon C, McFarland W (2013). Incorporating the service multiplier method in respondent-driven sampling surveys to estimate the size of hidden and hard-to-reach populations: case studies from around the world. Sex Transm Infect.

[82] Paz-Bailey G, Jacobson J, Guardado M, Hernandez F, Nieto A, Estrada M (2011). How many men who have sex with men and female sex workers live in El Salvador? Using respondent-driven sampling and capture–recapture to estimate population sizes. Sex Transm Infect.

[83] Quaye S, Raymond HF, Atuahene K, Amenyah R, Aberle-Grasse J, McFarland W (2015). Critique and lessons learned from using multiple methods to estimate population size of men who have sex with men in Ghana. AIDS Behav.

[84] Kelly GC, Tanner M, Vallely A, Clements A (2012). Malaria elimination: moving forward with spatial decision support systems. Trends Parasitol.

[85] Hustedt J, Canavati SE, Rang C, Ashton RA, Khim N, Berne L (2016). Reactive case-detection of malaria in Pailin Province, Western Cambodia: lessons from a year-long evaluation in a pre-elimination setting. Malar J.

[86] van Eijk AM, Ramanathapuram L, Sutton PL, Kanagaraj D, Sri Lakshmi Priya G, Ravishankaran S (2016). What is the value of reactive case detection in malaria control? A case-study in India and a systematic review. Malar J.

[87] Smith Gueye C, Sanders KC, Galappaththy GN, Rundi C, Tobgay T, Sovannaroth S (2013). Active case detection for malaria elimination: a survey among Asia Pacific countries. Malar J.

[88] Rosenberg NE, Pettifor AE, Bonogwe N, Mapanje C, Rutstein SE, Ward M (2014). STI patients are effective recruiters of undiagnosed cases of HIV: results of a social contact recruitment study in Malawi. J Acquir Immune Defic Syndr.

[89] Kimbrough LW, Fisher HE, Jones KT, Johnson W, Thadiparthi S, Dooley S (2009). Accessing social networks with high rates of undiagnosed HIV infection: the social networks demonstration project. Am J Public Health.

[90] Gouws E, Cuchi P (2012). Focusing the HIV response through estimating the major modes of HIV transmission: a multi-country analysis. Sex Transm Infect.

[91] Brown T, Bao L, Raftery AE, Salomon JA, Baggaley RF, Stover J (2010). Modelling HIV epidemics in the antiretroviral era: the UNAIDS Estimation and Projection package 2009. Sex Transm Infect.

[92] Stover J, Andreev K, Slaymaker E, Gopalappa C, Sabin K, Velasquez C (2014). Updates to the Spectrum model to estimate key HIV indicators for adults and children. AIDS.

[93] Griffin JT, Hollingsworth TD, Okell LC, Churcher TS, White M, Hinsley W (2010). Reducing *Plasmodium falciparum* malaria transmission in Africa: a model-based evaluation of intervention strategies. PLoS Med.

[94] Bertozzi SM, Laga M, Bautista-Arredondo S, Coutinho A (2008). Making HIV prevention programmes work. Lancet.

[95] The MEASURE Project (2004). The priorities for local AIDS control efforts (PLACE) manual.

[96] Weir SS, Pailman C, Mahlalela X, Coetzee N, Meidany F, Boerma JT (2003). From people to places: focusing AIDS prevention efforts where it matters most. AIDS.

[97] Heckathorn DD, Broadhead RS, Anthony DL, Weakliem DL (1999). AIDS and social networks: HIV prevention through network mobilization. Sociol Focus.

[98] Broadhead RS, Heckathorn DD, Grund JP, Stern LS, Anthony DL (1995). Drug users versus outreach workers in combating AIDS: preliminary results of a peer-driven intervention. J Drug Issues.

[99] Broadhead RS, Heckathorn DD, Weakliem DL, Anthony DL, Madray H, Mills RJ (1998). Harnessing peer networks as an instrument for AIDS prevention: results from a peer-driven intervention. Public Health Rep.

[100] Kim DA, Hwong AR, Stafford D, Hughes DA, O’Malley AJ, Fowler JH (2015). Social network targeting to maximise population behaviour change: a cluster randomised controlled trial. Lancet.

[101] Yan H, Zhang R, Wei C, Li J, Xu J, Yang H (2014). A peer-led, community-based rapid HIV testing intervention among untested men who have sex with men in China: an operational model for expansion of HIV testing and linkage to care. Sex Transm Infect.

[102] Smyrnov P, Broadhead RS, Datsenko O, Matiyash O (2012). Rejuvenating harm reduction projects for injection drug users: Ukraine’s nationwide introduction of peer-driven interventions. Int J Drug Policy.

[103] Ramos RL, Green NL, Shulman LC (2009). Pasa la Voz: using peer driven interventions to increase Latinas’ access to and utilization of HIV prevention and testing services. J Health Care Poor Underserved.

[104] Broadhead RS, Volkanevsky VL, Rydanova T, Ryabkova M, Borch C, van Hulst Y (2006). Peer-driven HIV interventions for drug injectors in Russia: first year impact results of a field experiment. Int J Drug Policy.

[105] Broadhead RS, Hammett TM, Kling R, Ngu D, Liu W, Chen Y (2009). Peer-driven interventions in Vietnam and China to prevent HIV: a pilot study targeting injection drug users. J Drug Issues.

[106] Tobgay T, Torres CE, Na-Bangchang K (2011). Malaria prevention and control in Bhutan: successes and challenges. Acta Trop.

[107] Gryseels C, Peeters Grietens K, Dierickx S, Xuan XN, Uk S, Bannister-Tyrrell M (2015). High mobility and low use of malaria preventive measures among the Jarai male youth along the Cambodia-Vietnam border. Am J Trop Med Hyg.

[108] Ministry of Health Malaysia, WHO, University of California (2015). Eliminating malaria: case-study 8—progress towards elimination in Malaysia.

[109] William T, Menon J (2014). A review of malaria research in Malaysia. Med J Malaysia.

[110] Ministry of Health Sri Lanka, WHO, University of California (2012). Eliminating malaria: case-sudy 3—progress towards elimination in Sri Lanka.

[111] Wangroongsarb P, Sudathip P, Satimai W (2012). Characteristics and malaria prevalence of migrant populations in malaria-endemic areas along the Thai-Cambodian border. Southeast Asian J Trop Med Public Health.

[112] Bhumiratana A, Sorosjinda-Nunthawarasilp P, Kaewwaen W, Maneekan P, Pimnon S (2013). Malaria-associated rubber plantations in Thailand. Travel Med Infect Dis.

[113] Pattanasin S, Satitvipawee P, Wongklang W, Viwatwongkasem C, Bhumiratana A, Soontornpipit P (2012). Risk factors for malaria infection among rubber tappers living in a malaria control program area in Southern Thailand. Southeast Asian J Trop Med Public Health.

[114] Erhart A, Ngo DT, Phan VK, Ta TT, Van Overmeir C, Speybroeck N (2005). Epidemiology of forest malaria in central Vietnam: a large scale cross-sectional survey. Malar J.

[115] Liu Y, Hsiang MS, Zhou H, Wang W, Cao Y, Gosling RD (2014). Malaria in overseas labourers returning to China: an analysis of imported malaria in Jiangsu Province, 2001–2011. Malar J.

[116] Xia Z, Yang M, Zhou S (2012). [Malaria situation in the People’s Republic of China in 2011] (in Chinese). Zhongguo Ji Sheng Chong Xue Yu Ji Sheng Chong Bing Za Zhi.

[117] Li Z, Yang Y, Xiao N, Zhou S, Lin K, Wang D (2015). Malaria imported from Ghana by returning gold miners, China, 2013. Emerg Infect Dis.

[118] Yin JH, Yang MN, Zhou SS, Wang Y, Feng J, Xia ZG (2013). Changing malaria transmission and implications in China towards National Malaria Elimination Programme between 2010 and 2012. PLoS ONE.

[119] Fighting malaria and TB in DPR Korea—a partnership approach. http://kp.one.un.org/health-in-dprk/.

[120] Barbieri AF, Sawyer IO, Soares-Filho BS (2005). Population and land use effects on malaria prevalence in the Southern Brazilian Amazon. Hum Ecol.

[121] Spencer BR. Gold mining and malaria in the Brazilian Amazon. Master’s Thesis, New Haven: Yale University; 1996.

[122] Vosti SA (1990). Malaria among gold miners in southern Para, Brazil: estimates of determinants and individual costs. Soc Sci Med.

[123] de Andrade ALS, Martelli CM, Oliveira RM, Arias JR, Zicker F, Pang L (1995). High prevalence of asymptomatic malaria in gold mining areas in Brazil. Clin Infect Dis.

[124] Adhin MR, Labadie-Bracho M, Vreden S (2014). Gold mining areas in Suriname: reservoirs of malaria resistance?. Infect Drug Resist.

[125] Venezuela: illegal mining and the resurgence of malaria. http://www.theguardian.com/global-development-professionals-network/2014/dec/02/valuing-amazonian-land-voices-tackling-malaria-venezuela.

[126] The malaria mines of Venezuela. http://www.bbc.com/news/health-28689066.

[127] Bashawri LA, Mandil AM, Bahnassy AA, Al-Shamsi MA, Bukhari HA (2001). Epidemiological profile of malaria in a university hospital in the Eastern Region of Saudi Arabia. Saudi Med J.

[128] Ministry of Health and Medical Industry of Turkmenistan, WHO, University of California (2012). Eliminating malaria: case-study 1—achieving elimination in Turkmenistan.

